# Design of Alkaline Earth‐Doped Co/MgO Catalysts for Ammonia Decomposition

**DOI:** 10.1002/cssc.202501801

**Published:** 2026-02-12

**Authors:** Sachika Hayashi, Yo Takeuchi, Takahiro Naito, K. Kanishka H. De Silva, Katsutoshi Sato, Takaaki Toriyama, Tomokazu Yamamoto, Yasukazu Murakami, Katsutoshi Nagaoka

**Affiliations:** ^1^ Department of Chemical Systems Engineering Graduate School of Engineering Nagoya University Nagoya Japan; ^2^ Institute of Innovation for Future Society Nagoya University Nagoya Japan; ^3^ Graduate School of Engineering Toyota Technological Institute Nagoya Japan; ^4^ Institute for Advanced Research Nagoya University Nagoya Japan; ^5^ The Ultramicroscopy Research Center Kyushu University Fukuoka Japan; ^6^ Department of Applied Quantum Physics and Nuclear Engineering Kyushu University Fukuoka Japan

**Keywords:** ammonia fuel, global warming, green energy conversion, H_2_ production, heterogeneous catalysis

## Abstract

Hydrogen is expected to be used as a fuel additive to ammonia, a non‐flammable and carbon‐free fuel, to improve combustion efficiency. However, the design strategies for developing highly active, nonprecious metal catalysts for ammonia decomposition are not yet well understood. Here, we show that Co/Ba_0.01_Mg_0.99_O exhibits high activity, with an ammonia conversion of 94.4% and a hydrogen production rate of 3.79 mol g_cat_^−1^ h^−1^ at 500°C with a WHSV of 60,000 mL g_cat_^−1^ h^−1^. Comparison of the dopant effects of alkaline earth metal elements elucidates that the high activity of Co/Ba_0.01_Mg_0.99_O is ascribed to the formation of a specific Co‐BaO core–shell‐like structure, with highly basic BaO nanoparticles covering the Co particles. The core–shell‐like structures were not formed with other alkaline earth elements. Such features facilitate efficient electron donation to Co nanoparticles, promoting N_2_ formation. Furthermore, kinetic analysis indicated that doping of alkaline earth metals weakens the adsorption of strongly bound species. Our findings will contribute to the development of cost‐effective supported metal catalysts for hydrogen production through ammonia decomposition, leading to the realization of a carbon‐neutral society in which ammonia plays a key role.

## Graphical abstract

Co/Ba_0_._01_Mg_0_._99_O exhibits high catalytic activity for H_2_ production via NH_3_ decomposition due to its Co‐BaO core–shell‐like structure, which does not occur with other alkaline earth elements. This architecture forms through reduction at 700°C, where MgO sintering induces Co nanoparticle migration. Migrating Co particles scrape melted Ba(OH)_2_ from the MgO surface, leading to BaO deposition on Co surfaces.

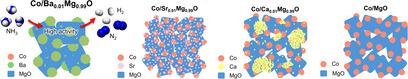

## 1 Introduction

Ammonia (NH_3_) is a fundamental chemical feedstock in various industrial processes and is produced worldwide at approximately 200 million tons per year [1]. Approximately 80% of the total volume is used as chemical fertilizers and their ingredients [2]. In addition, it has attracted much attention recently as a carbon‐free fuel for mitigating global warming [3]. This is because it combusts without the emission of CO_2_. In addition, ammonia is easily liquefied by simply compressing it at room temperature (25°C, 1 MPa), and has high hydrogen storage capacity (17.6 wt%) and high energy density (12.8 GJ m^−3^) in the liquid state, making it possible to transport hydrogen easily from the production site of renewable energy to consumption areas [4, 5].

Despite its usefulness, ammonia is difficult to burn. Thus, improving combustion efficiency is important [6]. It has been reported that several strategies can improve the combustion efficiency of ammonia: mixing ammonia with other fuels like hydrogen [7] or methane [8], or pre‐chamber ignition [9]. One method is to produce hydrogen by decomposing ammonia and then use this hydrogen as a combustion enhancer [10, 11]. Therefore, it is necessary to develop a catalyst that can decompose ammonia and produce hydrogen and nitrogen (Equation (1)) efficiently.

(1) $${\mathrm{NH}}_{3}\left(g\right)⇄1.5H_{2}\left(g\right)+0.5N_{2}\left(g\right)\Delta H=+46\;\mathrm{kJ}\;{\mathrm{mol}}^{‐1}$$

Lucentini et al. [12], Lee et al. [13], and Li et al. [14] reviewed the ammonia decomposition technology from the process and reactor perspectives. In addition, Zheng et al. [15] and Su et al. [16] reviewed the state‐of‐the‐art catalysts for ammonia decomposition. The kinds of catalysts that can be used include Ru‐, Fe‐, Co‐, or Ni‐based catalysts, supported on various materials, such as activated carbon, carbon nanotubes (CNTs), metal oxides, metal nitrides, metal carbides, and metal amides, and imides, etc [17, 18, 19, 20, 21, 22, 23]. Among them, supported Ru catalysts doped with strong basic compounds, such as alkaline and alkaline earth metal oxides, and having high electron conductivity and surface area, have shown excellent activity in ammonia decomposition [24, 25]. However, there is a need to establish guidelines for catalyst design using non‐precious metal catalysts, as Ru is one of the noble metal elements [26]. In addition to the choice of metal and support, promoters also play a crucial role in enhancing the NH_3_ decomposition efficiency. It has been reported that promoters with strong basicity, such as alkali and alkaline earth metal oxides, promote the NH_3_ decomposition rate due to their effective electronic modification ability [27, 28, 29].

Taking all these into consideration, in this research, we studied the effect of alkaline earth metal doping on Co/MgO for ammonia decomposition. MgO is one of the basic oxide supports with a high surface area [30]. The alkaline earth metal dopants used here, Ca, Sr, and Ba, possess lower electron negativity than Mg, thus improving the basic property of the Co/MgO catalyst. Since the Co/Ba_0.01_Mg_0.99_O catalysts showed the best activity, the cause was investigated using various characterization techniques, including STEM‐EDX, CO_2_‐TPD, and NH_3_‐TPSR. Our results show that strong basicity and the formation of BaO nanoparticles covering Co particles, which does not occur with other alkaline earth elements, are key to enhancing activity.

## 2 Results and Discussion

### 2.1 Ammonia Decomposition Activity of Co/MgO Promoted by Alkaline Earth Metals

**FIGURE 1:**
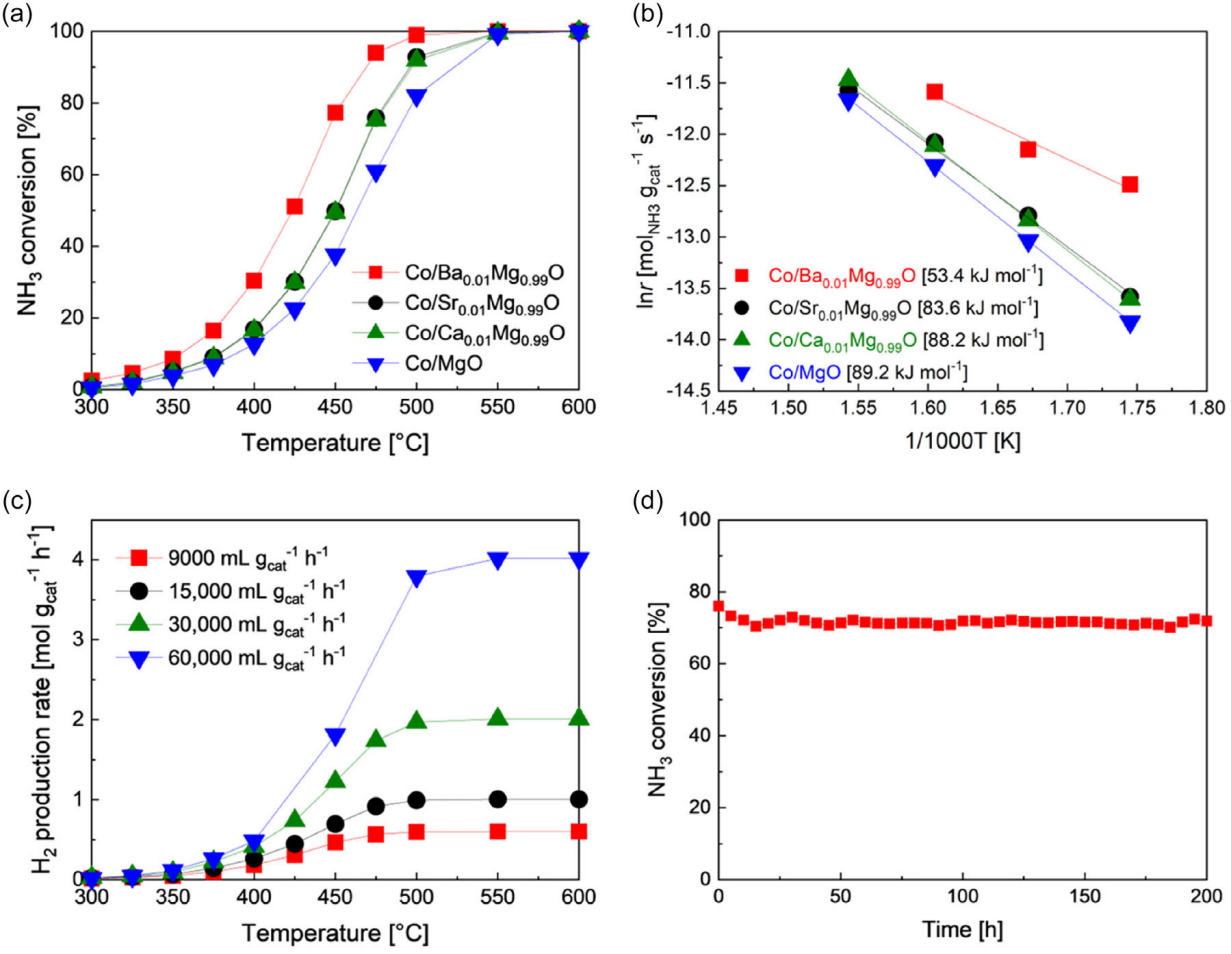
NH_3_ decomposition performance of Co‐based catalysts. (a) Temperature dependence of NH_3_ conversion of Co/MgO and Co/A_0.01_Mg_0.99_O (A = Ba, Sr, Ca) catalysts. WHSV of 9000 mL g_cat_^−1^ h^−1^. (b) Arrhenius plots for NH_3_ decomposition activity of Co‐based catalysts shown in (a). (c) Effect of WHSV on the H_2_ production rate of Co/Ba_0.01_Mg_0.99_O catalyst. (d) Stability tests of Co/Ba_0.01_Mg_0.99_O. Reaction conditions: WHSV of 9000 mL g_cat_^−1^ h^−1^ and 450°C.

Figure 1a shows NH_3_ conversion of Co/MgO and Co/MgO promoted with alkaline earth metals (Ca, Sr, and Ba) in the temperature range 300°C–600°C. All catalysts were reduced at 700°C in a H_2_ stream for 1 h before the reaction. The activity order of the Co catalysts was Co/Ba_0.01_Mg_0.99_O > Co/Sr_0.01_Mg_0.99_O = Co/Ca_0.01_Mg_0.99_O > Co/MgO, with the Co/Ba_0.01_Mg_0.99_O catalyst exceeding others and reaching nearly 100% at just 500°C. Meanwhile, the other Co catalysts approached 100% activity at 550°C. This trend aligns with the apparent activation energy estimated from Arrhenius plots (Figure 1b); Co/Ba_0.01_Mg_0.99_O < Co/Sr_0.01_Mg_0.99_O < Co/Ca_0.01_Mg_0.99_O < Co/MgO. The high activity of Co/Ba_0.01_Mg_0.99_O is attributed to its low apparent activation energy. We examined how Ba doping amount affects performance, with results shown in Figure S1. NH_3_ conversion rose sharply with 0.5 mol% Ba doping to Co/MgO between 300°C and 500°C. Doping of Ba up to 1 mol% maintained a high activity, but further doping until 2 mol% led to a decrease in NH_3_ conversion. Therefore, we used Co/MgO doped with 1 mol% Ba (Co/Ba_0.01_Mg_0.99_O), for further tests. Next, we assessed the influence of Co loading on Co/Ba_0.01_Mg_0.99_O catalysts (Figure S2). Increasing Co loading from 10% to 20% improved the NH_3_ conversion, but further increases to 30% had a limited effect. Thus, we chose 20 wt% Co/Ba_0.01_Mg_0.99_O for additional studies.

Figure 1c depicts the effect of WHSV on the H_2_ production rate of Co/Ba_0.01_Mg_0.99_O catalyst. Accordingly, the rate increased with the increase in WHSV from 9000 to 60,000 mL g_cat_^−1^ h^−1^. A stable NH_3_ decomposition rate at 500°C–600°C indicates that NH_3_ conversion approached 100%. We compared NH_3_ decomposition rates of Co/Ba_0.01_Mg_0.99_O with other Co catalysts reported in the literature (Table S1). Although it is difficult to compare the activity directly among the catalysts measured in the different reactors and different reaction conditions, Co/Ba_0.01_Mg_0.99_O shows a higher or comparable NH_3_ decomposition rate to those of other Co and Ni catalysts. Stability tests were conducted at 450°C using the optimal Co/Ba_0.01_Mg_0.99_O catalyst. This was because, although the conversion rate of NH_3_ was high at this temperature, it did not reach 100%. As shown in Figure 1d, the catalyst maintained its initial NH_3_ conversion for 200 h, revealing its excellent stability during NH_3_ decomposition. Although Chorkendorff et al. reported that Co/Ba/C catalyst has demonstrated high activity in NH_3_ decomposition, the presence of carbon raises stability concerns under the high‐temperature conditions required to reduce NH_3_ concentrations to ppm levels. By contrast, Ba_0.01_Mg_0.99_O catalysts use an oxide support, which resolves these stability issues [31].

### 2.2 Kinetic Analysis

Kinetic analysis was performed at 400°C. The reaction rate (*r*) was described as Equation (2) using the reaction rate constant (*k*), the partial pressures of ammonia (*P*_NH3_), nitrogen (*P*_N2_), and hydrogen (*P*_H2_), and their respective reaction orders *α*, *β*, and *γ*:

(2) $$r=k{P_{{\mathrm{NH}}_{3}}}^{\alpha }{P_{N_{2}}}^{\beta }{P_{H_{2}}}^{\gamma }$$

Reaction orders were estimated using reaction rates obtained by varying the partial pressure of each gas, and the results are shown in Table 1. First, the reaction orders for ammonia (*α*) were estimated to be 0.98 for Co/Ba_0.01_Mg_0.99_O, 0.92 for Co/MgO, and minimized at 0.78–0.79 for Co/Sr_0.01_Mg_0.99_O and Co/Ca_0.01_Mg_0.99_O, showing no correlation with activity. Second, the reaction orders of N_2_ (*β*) were found to be approximately zero for all catalysts, indicating that the partial pressure of nitrogen does not affect the reaction rate. Third, the reaction orders for hydrogen (*γ*) were −1.05 for Co/MgO, while other catalysts showed higher values, indicating that the adsorption of strongly adsorbed species (H, NH_3_, NH_2_, or NH) was weakened by the doping of alkaline earth metals. However, as mentioned in previous reports [32, 33], it is known that these reaction orders can also be obtained using kinetic models that consider different rate‐determining steps, such as those assuming dehydrogenation of NH_*x*_ (*x* = 1–3) or N–Co dissociation. Therefore, the rate‐limiting step could not be determined.

**TABLE 1:** Reaction order for NH_3_, N_2_, and H_2_ estimated at 400°C, WHSV of 9000 mL g_cat_^−1^ h^−1^ over Co/A_0.01_Mg_0.99_O (A = Ba, Sr, Ca) and Co/MgO catalysts.

Catalyst	*α*	*β*	*γ*
Co/Ba_0.01_Mg_0.99_O	0.98	−0.02	−0.79
Co/Sr_0.01_Mg_0.99_O	0.78	0.00	−0.72
Co/Ca_0.01_Mg_0.99_O	0.79	−0.01	−0.74
Co/MgO	0.92	−0.02	−1.05

### 2.3 Characterization of Co Catalysts

**FIGURE 2:**
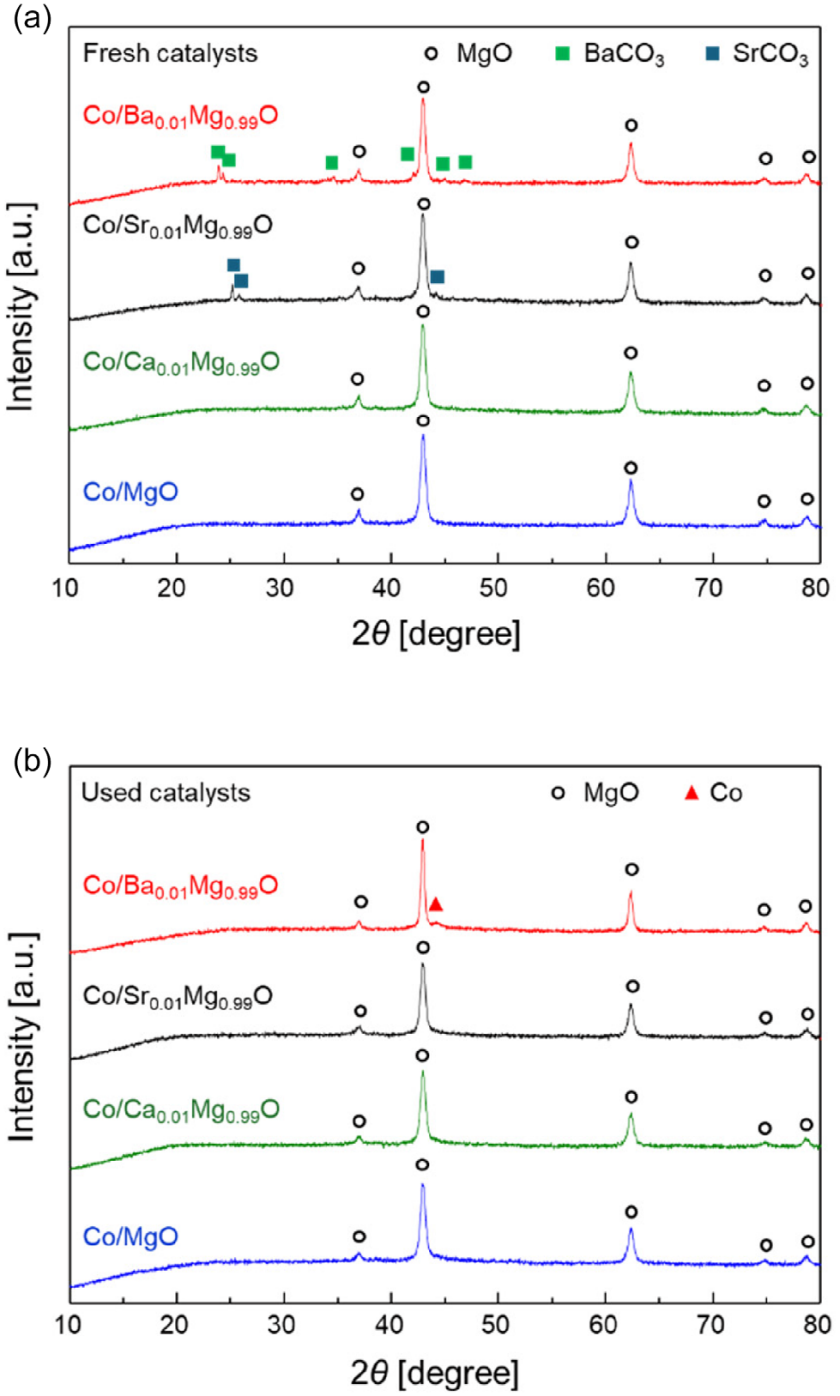
XRD patterns of CoA_0.01_Mg_0.99_O (A = Ba, Sr, Ca) and Co/MgO. (a) Fresh catalysts. (b) Used catalysts.

Figure 2 shows the XRD patterns of the supported Co catalysts promoted with various alkaline earth metals. For the Co/Ba_0.01_Mg_0.99_O catalyst, we confirmed that there is no change between the XRD patterns of the reduced (before reaction) and used (after reaction) catalysts, because the H_2_ reduction temperature was sufficiently high (700°C) compared with the maximum temperature used for the reaction (600°C). The peaks corresponding to MgO were observed in all the fresh catalysts. In addition, the formation of alkaline earth carbonates, such as BaCO_3_ and SrCO_3_ was observed only in the fresh Co/Ba_0.01_Mg_0.99_O and Co/Sr_0.01_Mg_0.99_O catalysts, indicating hydrogenation of the carbonates occurred during reduction at 700°C. We speculate we did not observe CaCO_3_ in the Ca‐doped fresh catalyst, probably due to low crystallinity of the compound. In addition, a small peak ascribed to Co nanoparticles was observed for the used Co/Ba_0.01_Mg_0.99_O, although it was not observed for the other used catalysts. These results indicate that Co nanoparticles are sintered in the Co/Ba_0.01_Mg_0.99_O catalyst. Moreover, according to the BET specific surface area results (Table 1), the used Co/Ba_0.01_Mg_0.99_O catalyst has the smallest value among the others. This can be due to the sintering of the MgO support. Hence, it can be assumed that the sintering of Co nanoparticles is associated with the sintering of MgO support. These results were further confirmed by STEM images together with EDX elemental maps (see later section).

The H_2_ chemisorption capacity of Co catalysts is summarized in Table 2. With the doping of Sr and Ca to Co/MgO, the capacity was increased, but the doping of Ba resulted in a drastic decrease of 56%. In contrast, the TOF of the Co/Ba_0.01_Mg_0.99_O catalyst is the highest and about three times that of other Co catalysts. Doping of Sr and Ca also increased the TOF slightly compared with Co/MgO. Therefore, it was found that high TOF contributes to high NH_3_ conversion of the Co/Ba_0.01_Mg_0.99_O catalyst, although the Co surface area decreased by Ba doping.

**TABLE 2:** Physicochemical properties and catalytic performance at 450°C of Co catalysts.

Catalyst	Specific surface area, m^2^ g_cat_^−1^		H_2_ chemisorption, µmol g_cat_^−1^	NH_3_ conversion, %	H_2_ production rate, mol g_cat_^−1^ h^−1^	TOF, s^−1^
	fresh	used				
Co/Ba_0.01_Mg_0.99_O	85	41	36	74	0.45	1.15
Co/Sr_0.01_Mg_0.99_O	91	70	70	49	0.30	0.40
Co/Ca_0.01_Mg_0.99_O	97	71	79	50	0.30	0.36
Co/MgO	104	78	64	37	0.22	0.32

**FIGURE 3:**
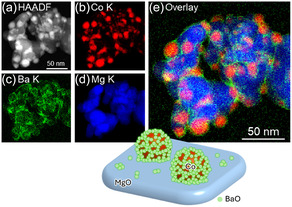
HAADF–STEM images, EDX maps, and a schematic image of used Co/Ba_0.01_Mg_0.99_O catalyst. (a) HAADF image. (b–e) EDX maps. (e) Overlay EDX map of (b) Co K, (c) Ba K, and (d) Mg K.

**FIGURE 4:**
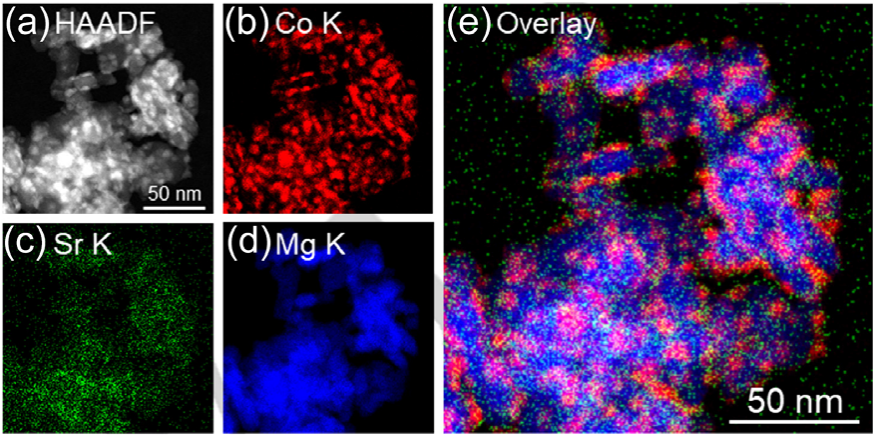
HAADF–STEM images and EDX maps of used Co/Sr_0.01_Mg_0.99_O catalyst. (a) HAADF image. (b–e) EDX maps. (e) Overlay EDX map of (b) Co K, (c) Sr K, and (d) Mg K.

**FIGURE 5:**
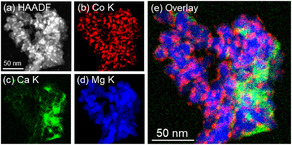
HAADF–STEM images and EDX maps of used Co/Ca_0.01_Mg_0.99_O catalyst. (a) HAADF image. (b–e) EDX maps. (e) Overlay EDX map of (b) Co K, (c) Ca K, and (d) Mg K.

**FIGURE 6:**
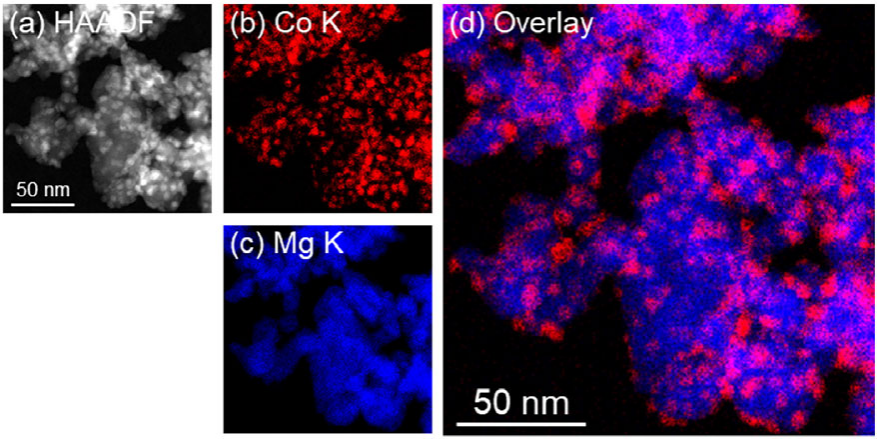
HAADF–STEM images and EDX maps of used Co/MgO catalyst. (a) HAADF image. (b–d) EDX maps. (d) Overlay EDX map of (b) Co K and (c) Mg K.

Figures 3–6 show HAADF–STEM images and corresponding EDX elemental maps of used Co catalysts, and the size distribution of Co nanoparticles supported on each catalyst is displayed in Figure S3. According to Figure 3, large Co particles with a mean diameter of 11.7 ± 3.1 nm were observed for used Co/Ba_0.01_Mg_0.99_O, confirming the results obtained by XRD measurements. Moreover, Ba was enriched over the surface of Co nanoparticles, resembling core–shell‐like structures. As given in Figure 4, Co nanoparticles with a mean diameter of 5.7 ± 1.5 nm were observed for Co/Sr_0.01_Mg_0.99_O, and Sr was dispersed throughout the catalyst. On the other hand, small Co nanoparticles with a mean diameter of 6.2 ± 1.4 nm are observed for Co/Ca_0.01_Mg_0.99_O, and Ca was segregated from the catalyst but was not enriched over Co nanoparticles like in the Co/Ba_0.01_Mg_0.99_O catalyst (Figure 5). Note that Ca segregation occurred prior to reduction (Figure S4) and persisted during reduction (Figure S5) and reaction. Figure 6 depicts the STEM‐EDX images of the nonpromoted Co/MgO catalyst. Accordingly, small Co nanoparticles with a mean diameter of 5.2 ± 1.1 nm were observed. Overall, the STEM‐EDX results elucidate that only Ba is enriched over relatively large Co nanoparticles. Surface coverage of large Co particles by BaO well explains the low H_2_ chemisorption capacity of the Co/Ba_0.01_Mg_0.99_O catalyst. On the other hand, since Sr or Ca was not enriched over Co nanoparticles, H_2_ chemisorption over these catalysts was rather increased than Co/MgO, which implies a decrease in Co particle size. The discrepancy in particle size between H_2_ chemisorption and STEM is probably due to differences in analytical methods. It is important to understand why only Ba makes core–shell‐like structures with Co among different alkaline earth dopants. This is in agreement with our previous report, which elucidates that BaO nano‐fractions cover the surface of Co nanoparticles during reduction at 700°C [30]. In brief, BaCO_3_, which is present in the fresh catalyst, is converted to Ba(OH)_2_ with a low melting point (408°C) during H_2_ reduction, and this liquified Ba(OH)_2_ covers the surface of Co nanoparticles to decrease their surface energy as they move across MgO at high temperatures. The Ba(OH)_2_ is dehydrated to BaO, which coagulates as nanofractions on Co nanoparticles due to its high melting point (1920°C). On the other hand, we did not observe CaCO_3_ in the Ca‐doped fresh catalyst, probably due to the low crystallinity of the compound. Since the melting points of Ba(OH)_2_, Sr(OH)_2_, and Ca(OH)_2_ are 408°C, 385°C, and 580°C, respectively, liquefaction occurs for all hydroxides during H_2_ reduction at 700°C. These hydroxides then decomposed to oxides during the H_2_ reduction. In addition, the melting points of BaO, SrO, and CaO are 1923°C, 2430°C, and 2572°C, respectively, so coagulation may proceed over these catalysts. On the other hand, STEM analysis as well as XRD patterns of the used catalysts clearly show that the Co nanoparticle size has increased only in the Ba‐promoted catalyst. This indicates that the liquefied Ba(OH)_2_ was scraped off by the Co nanoparticles that moved on the MgO support, simultaneously with sintering of both the Co nanoparticles and the MgO support during the reduction from 500°C to 700°C. However, the reason why this phenomenon occurs exclusively with Co/Ba_0.01_Mg_0.99_O catalyst remains unclear and warrants further investigation.

**FIGURE 7:**
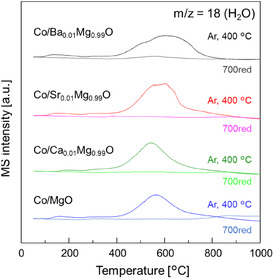
H_2_‐TPR profiles of Co/A_0.01_Mg_0.99_O (A = Ba, Sr, Ca) and Co/MgO after Ar treatment at 400°C and H_2_ reduction at 700°C: *m*/*z* = 18.

The reduction degree of the active metal is one of the important factors for the catalytic activity. Therefore, H_2_‐TPR measurements were performed for the fresh catalysts and after reduction at 700°C (Figure 7). For all fresh catalysts, after Ar treatment at 400°C, H_2_O formation peak ascribed to the reduction of Co oxide was observed. A maximum peak at around 550°C was seen for Co/Ca_0.01_Mg_0.99_O and Co/MgO catalysts, which was shifted to higher temperatures for Co/Sr_0.01_Mg_0.99_O and even higher for Co/Ba_0.01_Mg_0.99_O. Notably, no peak was observed for all catalysts after reduction at 700°C, indicating that Co oxides were almost reduced during the pre‐reduction.

**FIGURE 8:**
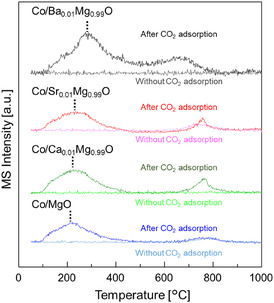
CO_2_‐TPD profiles (*m*/*z* = 44) of Co/A_0.01_Mg_0.99_O (A = Ba, Sr, Ca) and Co/MgO after or without CO_2_ adsorption at 50°C.

The CO_2_‐TPD profiles were measured to evaluate the basicity of the Co catalysts (Figure 8). Here, CO_2_ desorption profiles normalized by specific surface area are displayed. At first, CO_2_ TPD was performed without CO_2_ adsorption for the reduced Co catalysts. For Co/Sr_0.01_Mg_0.99_O and Co/MgO, small peaks were observed at about 750°C, which are ascribed to carbonate remaining after H_2_ reduction. This further indicates that some of the SrCO_3_ and MgCO_3_ in these catalysts might not have been completely reduced, which may lead to a decrease in the basicity of these catalysts. The difference between the TPD profiles with and without CO_2_ adsorption can be related to the basicity of the catalyst. For all catalysts, CO_2_ desorption peaks were observed within the temperature range of 100°C–500°C, and the peak corresponding to maximum desorption shifted toward higher temperatures as the electron negativity of the dopant elements decreased (electron negativity; Ca > Sr > Ba). A small peak (700°C–900°C) indicating the presence of strong basic sites or decomposition of stable carbonates was observed for Co/Ca_0.01_Mg_0.99_O and Co/Sr_0.01_Mg_0.99_O. On the other hand, CO_2_ desorption was observed over a wide temperature range (100°C–800°C) for Co/Ba_0.01_Mg_0.99_O, which indicates the formation of relatively strong basic sites related to a core–shell‐like structure. Furthermore, the total amount of basic sites (total area of peaks) per specific surface area was highest for Co/Ba_0.01_Mg_0.99_O among all the catalysts. Therefore, the proximity of BaO as a nanofraction to Co nanoparticles, as well as its strong basicity, maximizes electron donation from BaO to Co nanoparticles. In the case of Sr or Ca‐doped catalysts, only a strong basic property contributes to donating electrons to Co nanoparticles.

**FIGURE 9:**
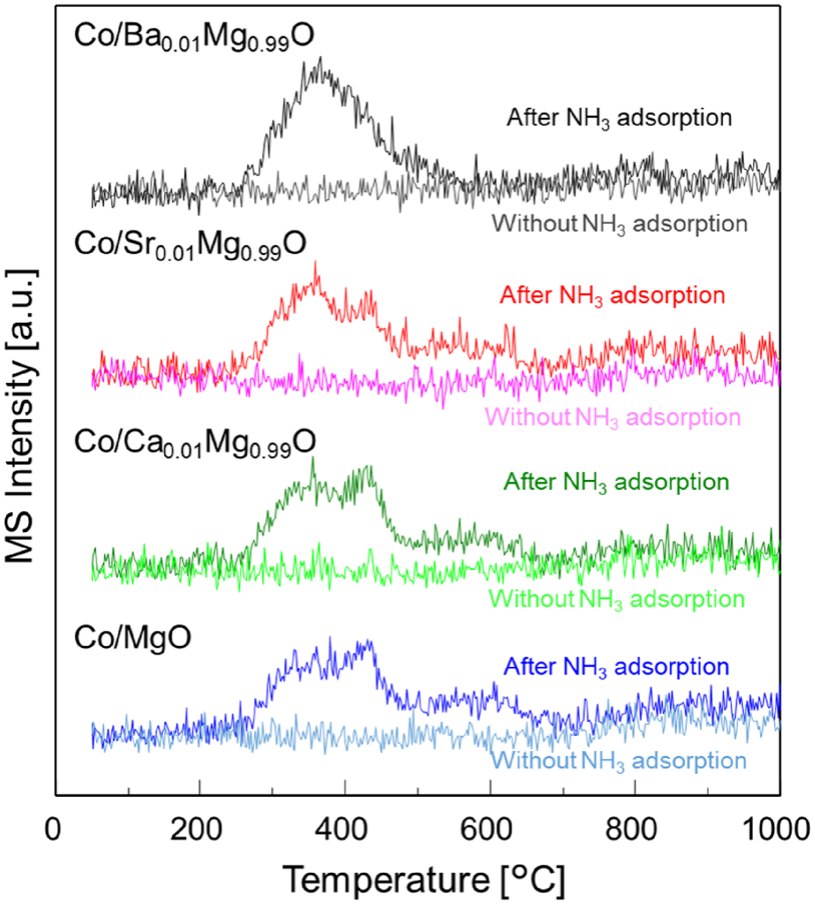
NH_3_‐TPSR profiles of Co/A_0.01_Mg_0.99_O (A = Ba, Sr, Ca) and Co/MgO after or without NH_3_ adsorption at 400°C. N_2_ (*m*/*z* = 14).

To understand the N_2_ desorption step, NH_3_‐TPSR was examined after NH_3_ adsorption at 400°C. First, NH_3_‐TPSR was performed without NH_3_ adsorption for the reduced Co catalysts. The difference between the NH_3_‐TPSR profiles with and without NH_3_ adsorption is related to the desorption after NH_3_ adsorption. For all catalysts, NH_3_ desorption was observed from room temperature to 600°C (Figure S4). Next, for all catalysts, N_2_ desorption began at around 250°C (Figure 9). For Co/Ba_0.01_Mg_0.99_O, the desorption peak reached its maximum at 365°C, and completed below 560°C. On the other hand, Co/Sr_0.01_Mg_0.99_O and Co/Ca_0.01_Mg_0.99_O catalysts showed three peaks with peak maxima at around 360°C, 430°C, and 560°C, and N_2_ desorption continued up to 660°C. Furthermore, the Co/MgO catalyst showed a similar profile to that of Co/Sr_0.01_Mg_0.99_O and Co/Ca_0.01_Mg_0.99_O catalysts, and N_2_ desorption completed at about 720°C. These results reveal that the NH_*x*_ dehydrogenation or the N‐Co dissociation, which is an important step for N_2_ formation, is facilitated most effectively over Co/Ba_0.01_Mg_0.99_O and slightly over Co/Sr_0.01_Mg_0.99_O and Co/Ca_0.01_Mg_0.99_O compared with Co/MgO. In brief, electron donation from BaO nanoparticles covering Co particles with strong basic properties promotes this step. This accounts for the low apparent activation energy of the Co/Ba_0.01_Mg_0.99_O catalyst (Figure 1b).

## 3 Conclusions

We have revealed that Co/Ba_0.01_Mg_0.99_O, consisting of a Co‐BaO core–shell‐like structure, shows high and stable activity for hydrogen production by ammonia decomposition. Our systematic study revealed that this structure is obtained by reduction of Co/Ba_0.01_Mg_0.99_O at 700°C. Sintering of MgO induces the migration of Co nanoparticles, which in turn scrape off the melted Ba(OH)_2_ from the MgO surface. This process leads to the deposition of BaO on the surface of the Co nanoparticles, resulting in a core–shell‐like structure. The accumulated BaO with strong basic property donates electrons to the Co nanoparticles and promotes N_2_ formation. In contrast, such a core–shell‐like structure was not formed for Co/Sr_0.01_Mg_0.99_O and Co/Ca_0.01_Mg_0.99_O. Thus, only the electron donation effect from SrO and CaO with medium basic property promotes ammonia decomposition moderately. Furthermore, kinetic analysis indicated that the adsorption of strongly bound species was weakened by the doping of alkaline earth metals. Our findings provide a strategy for the effective design of supported metal catalysts and will contribute to the realization of ammonia and hydrogen fuel power plants, which are required in a carbon‐neutral society.

## 4 Experimental Section

### 4.1 Catalysis Preparation

Co catalysts doped with various alkaline earth metals (A = Ba, Sr, and Ca) and supported on MgO were prepared by sequential impregnation. First, the oxide support was prepared by mixing MgO (MgO‐500A, Ube Material Industries, Japan) and alkaline earth metal hydroxides (A(OH)_2_). Briefly, 5 g MgO was dispersed in ultrapure water and then heated at 70°C with stirring until the water evaporated. 200 mL of aqueous solutions of the alkaline earth metal hydroxides were separately prepared by stirring for 1 h, and 2.49 g of MgO powder was mixed with the hydroxide solutions by stirring for 1 h so that the molar percentage of ‘A’ is 1% with respect to A + Mg. The mixtures were then dried using the rotary evaporator and calcined in a furnace at 700°C for 5 h in static air. Next, 1 g of these supports was impregnated with 1.36 g of cobalt (II) acetylacetonate dihydrate [Co(acac)_2_·2H_2_O] (Fujifilm Wako) in 200 mL of tetrahydrofuran (Fujifilm Wako) solvent, kept for 18 h, and dried using the rotary evaporator at 35°C. The Co loading was set to 20% by weight of the catalyst. The resulting powder was dried in an oven at 80°C for 18 h and then heated in a tubular furnace at 500°C for 5 h under Ar flow to remove the ligand from Co(acac)_2_. Additionally, a Co/MgO catalyst without alkaline earth metals was prepared using the same procedure. These catalysts were denoted as Co/A_0.01_Mg_0.09_O, where A = Ba, Sr, and Ca. A series of catalysts was prepared by changing the molar ratio of Ba between 0.5% and 2% and another series was prepared by changing the Co wt% in the Ba_0.01_Mg_0.09_O catalyst.

### 4.2 Catalytic Activity Test

A fixed‐bed flow system was used to measure ammonia decomposition activity. 0.1 g of catalyst in pellet form (250–500 µm) was placed in a quartz reaction tube, and the tip of a thermocouple was fixed in place so that it is in the middle of the catalyst bed. For pretreatment, the catalyst was reduced at 700°C for 1 h under pure hydrogen (60 mL min^−1^). After that, NH_3_ (15 mL min^−1^, WHSV = 9000 mL g_cat_^−1^ h^−1^) was introduced at atmospheric pressure at 300°C. In the activity measurement, the reaction temperature was set to 300°C–700°C. The NH_3_ conversion rate was calculated by quantifying H_2_, N_2_, and NH_3_ using a system gas chromatograph equipped with a thermal conductivity detector (GL Sciences Inc., Japan). NH_3_ conversion was calculated using Equations (2) and (3). To improve the accuracy of the analysis, Equation (2) was used when the NH_3_ conversion rate was 7% or more, and Equation (3) was used when it was less than 7%. When changing the weight hourly space velocity (WHSV), the NH_3_ flow rate was adjusted.

(3) $${\mathrm{NH}}_{3}\;\text{conversion}\left[\%\right]=\frac{100-{\mathrm{NH}}_{3}\;\text{concentration}(\%)}{100+{\mathrm{NH}}_{3}\;\text{concentration}(\%)}\times 100$$

(4) $${\mathrm{NH}}_{3}\;\text{conversion}\left[\%\right]=\frac{N_{2}\;\text{concentration}(\%)}{50-N_{2}\;\text{concentration}(\%)}\times 100$$

In the kinetic analysis, reaction orders were determined using reaction rates obtained by varying the partial pressure of each gas. As with the activity tests, the same fixed‐bed flow system and 0.1 g of catalyst were used, and the reaction temperature was set to 400°C. For determining the reaction order with respect to nitrogen, the ammonia concentration was set at approximately 60%, and the partial pressures of nitrogen and He were varied. For determining the reaction rate order with respect to ammonia, the partial pressure of hydrogen was set at 33.3%, and the partial pressures of ammonia and He were varied. Furthermore, for determining the reaction order with respect to hydrogen, the ammonia concentration was set at 33.3%, and the partial pressures of hydrogen and He were varied.

### 4.3 Characterization

The crystal structure of the catalysts was analyzed by X‐ray diffraction (XRD) measurements using a Smart‐Lab X‐ray diffractometer (Rigaku, Japan) with a Cu K_*α*_ source. The XRD patterns were analyzed using the PDXL2 software (Rigaku) with ICDD and COD [34] databases. The crystallite size of Co^0^ was estimated by Scherrer's equation using the Co^0^ 111 peak located at 44.2° in the XRD patterns.

To evaluate the turnover frequency (TOF) of the reduced catalyst, H_2_ chemisorption capacity was estimated by H_2_ pulse injection method. 0.1 g of each catalyst was packed in a quartz tube in BELCAT II (MicrotracBEL, Japan). The H_2_ reduction was carried out in situ at 700°C for 1 h under a gas flow of 60 mL min^−1^, after which the feed gas was switched to Ar and held for 2 h. After that, the catalyst bed temperature was lowered to 50°C in Ar, and H_2_ gas was pulsed to the catalyst under the Ar flow. The pulse was continued until no H_2_ consumption was observed. It was assumed here that adsorption occurs at H/Co = 1.

Temperature‐programmed reduction under a pure H_2_ flow (H_2_‐TPR) was performed using BELCAT II. For the TPR measurement of the fresh catalyst before reduction, 0.1 g of the catalyst was packed into the reactor and heated at 400°C for 1 h to remove adsorbed impurities and water. After cooling to 50°C under Ar flow, the temperature was increased to 1000°C at a rate of 10°C min^−1^ under pure H_2_ flow. The TPR measurement of the catalyst after reduction was carried out as follows. The sample was reduced at 700°C for 1 h under a flow of H_2_ at 60 mL min^−1^, followed by purging with Ar for 2 h. After that, the temperature was lowered to 50°C under Ar flow, then gas was switched to a H_2_ flow, and sample was heated to 1000°C at a rate of 10°C min^−1^. Produced species during the temperature increment was monitored by the quadruped mass spectrometer (Q‐MS, BELMASS, MicrotracBEL).

High‐angle annular dark‐field scanning transmission electron microscope (HAADF–STEM) images and energy dispersive X‐ray (EDX) elemental maps were obtained using an aberration‐corrected scanning transmission electron microscope (JEM‐ARM200CF, JEOL) equipped with an EDX detector. Samples were dispersed in ethanol at room temperature, dropped onto a carbon‐coated copper grid, and vacuum dried for 24 h at room temperature prior to measurements. Mean diameter of Co nanoparticles supported on each catalyst was estimated by HAADF–STEM images.

The specific surface area (SSA) of each sample was measured according to Brunauer–Emmett–Teller theory with a BELSORP MINI X (MicrotracBEL).

Temperature‐programmed desorption of CO_2_ (CO_2_‐TPD) measurements were carried out using a BELCAT II. 0.1 g of the sample was placed in a quartz tube, then the sample was reduced at 700°C for 1 h under a H_2_ flow of 60 mL min^−1^, and then held for 1 h under a He atmosphere. The temperature was then lowered to 50°C while maintaining the He atmosphere, and then 5.0% CO_2_/He was flowed at 50 mL min^−1^ for 30 min to adsorb CO_2_ onto the sample. The sample was then heated to 1000°C while He gas was passed through it, and the desorption behavior of CO_2_ was observed using a Q‐MS (BELMASS). To confirm that the observed CO_2_ was derived from the introduced CO_2_, blank‐TPD was also performed by the same procedure except for the step of introducing CO_2_.

Temperature‐programmed surface reaction with NH_3_ (NH_3_‐TPSR) measurements were carried out using BELCAT II. First, 0.1 g of the sample was placed in a quartz tube and reduced at 700°C for 1 h under a H_2_ flow (60 mL min^−1^), followed by treatment under He flow for an additional 1 h. Subsequently, the temperature was lowered to 400°C under a He atmosphere, and pure NH_3_ was introduced at 50 mL min^−1^ for 1 h to allow adsorption onto the sample. After NH_3_ adsorption, the temperature was further lowered to 50°C while maintaining the NH_3_ atmosphere. The sample was then heated to 1000°C while He gas was passed through it, and the NH_3_ (*m*/*z* = 17), H_2_ (*m*/*z* = 2), and N_2_ (*m*/*z* = 14) formation was observed using a Q‐MS (BELMASS). For comparison, the NH_3_‐TPSR was also performed without supplying NH_3_.

## Supporting Information

Additional supporting inforamtion can be found online in the Supporting Inforamtion section. The authors have cited additional references within the Supporting Information [4, 25, 31, 35, 36, 37, 38, 39, 40, 41, 42, 43, 44, 45]. **Supporting** **Fig.** **S1:** Influence of Ba amount on the activity of Co/Ba_0.01_Mg_0.99_O catalyst. WHSV of 9000 mL g_cat_^−1^ h^−1^. **Supporting** **Fig.** **S2:** Influence of Co loading on the activity of Co/ Ba_0.01_Mg_0.99_O catalyst. WHSV of 9000 mL g_cat_^−1^ h^−1^. **Supporting** **Fig.** **S3:** HAADF STEM images and Co nanoparticle distributions for Co/ Ba_0.01_Mg_0.99_O (a–b), Co/ Sr_0.01_Mg_0.99_O (c–d), Co/ Sr_0.01_Mg_0.99_O (e–f), Co/MgO (g–h). **Supporting** **Fig.** **S4:** HAADF‐STEM images and EDX maps of fresh Co/Ca_0.01_Mg_0.99_O catalyst. (a) HAADF image. (b–e) EDX maps. (e) Overlay EDX map of (b) Co K, (c) Ca K, and (d) Mg K. **Supporting** **Fig.** **S5:** HAADF‐STEM images and EDX maps of Co/Ca_0.01_Mg_0.99_O catalyst after reduction. (a) HAADF image. (b‐e) EDX maps. (e) Overlay EDX map of (b) Co K, (c) Ca K, and (d) Mg K. **Supporting** **Fig.** **S6:** NH_3_‐TSR profiles of Co/A_0.01_Mg_0.99_O (A = Ba, Sr, Ca) and Co/MgO after or without NH_3_ adsorption at 400°C. NH_3_ (m/z = 17). **Supporting** **Table** **S1:** Comparison of the catalytic activity of Co‐based catalysts reported by other group.

## Author Contributions

**Sachika Hayashi**: data curation (lead), formal analysis (equal), investigation (equal), writing – original draft (equal), writing – review and editing (supporting). **Yo Takeuchi**: data curation (supporting), formal analysis (supporting), investigation (supporting), writing – review and editing (supporting). **Takahiro Naito**: investigation (supporting), validation (supporting), writing – review & editing (equal). **K. Kanishka H. De Silva**: investigation (equal), validation (supporting), writing – original draft (equal), writing – review and editing (equal). **Katsutoshi Sato**: formal analysis (equal), investigation (equal), methodology (lead), supervision (equal), validation (equal), writing – original draft (supporting), writing – review and editing (equal). **Takaaki Toriyama**: data curation (equal), investigation (equal), validation (equal), writing – review and editing (equal). **Tomokazu Yamamoto**: data curation (equal), formal analysis (equal), investigation (equal), methodology (equal), validation (equal), writing – review and editing (equal). **Yasukazu Murakami**: data curation (equal), formal analysis (equal), investigation (equal), methodology (equal), supervision (equal), validation (equal), writing – review and editing (equal). **Katsutoshi Nagaoka**: conceptualization (lead), formal analysis (equal), funding acquisition (lead), investigation (lead), methodology (equal), supervision (lead), validation (lead), writing – original draft (equal), writing – review and editing (lead).

## Funding

This work was supported by the Ministry of Education, Culture, Sports, Science and Technology (JP) (JPMXP1224KU0019 and JPMXP1225KU0023) and Fusion Oriented REsearch for disruptive Science and Technology (JPMJFR223N).

## Conflicts of Interest

The authors declare no conflicts of interest.

## Supporting information

Supplementary Material

## Data Availability

The data that support the findings of this study are available on request from the corresponding author. The data are not publicly available due to privacy or ethical restrictions.
