# Supplementary material for: Design of Alkaline Earth‐Doped Co/MgO Catalysts for Ammonia Decomposition
Source: ChemSusChem. 2026 Feb 12;19(4):e202501801. doi: 10.1002/cssc.202501801 (PMC12900564; doi:10.1002/cssc.202501801)
Supplement: Supplementary file 1 — Supplementary Material [file CSSC-19-e202501801-s001.pdf]

Supporting Information for

Design of Alkaline Earth-Doped Co/MgO Catalysts for Ammonia Decomposition

Sachika Hayashi[1], Yo Takeuchi[1], Takahiro Naito[2], K. Kanishka H. De Silva[2], Katsutoshi Sato[1,3]*, Takaaki Toriyama[4], Tomokazu Yamamoto[4], Yasukazu Murakami[4,5], Katsutoshi Nagaoka[1,2]*

1) Department of Chemical Systems Engineering, Graduate School of Engineering, Nagoya University, Furo-cho, Chikusa-ku, Nagoya,464-8603, Japan
2) Institute of Innovation for Future Society, Nagoya University, Furo-cho, Chikusa-ku, Nagoya,464-8603, Japan
3) Institute for Advanced Research, Nagoya University, Furo-cho, Chikusa-ku, Nagoya, Aichi 464-8601, Japan
4) The Ultramicroscopy Research Center, Kyushu University, Motooka 744, Nishi-ku, Fukuoka 819-0395, Japan.
5) Department of Applied Quantum Physics and Nuclear Engineering, Kyushu University, Motooka 744, Nishsi-ku, Fukuoka 819-0395, Japan.

†Present address Dr. K. Kanishka H. De Silva
Graduate School of Engineering
Toyota Technological Institute
Hisakata 2-12-1, Tenpaku, Nagoya, Aichi 468-8511, Japan

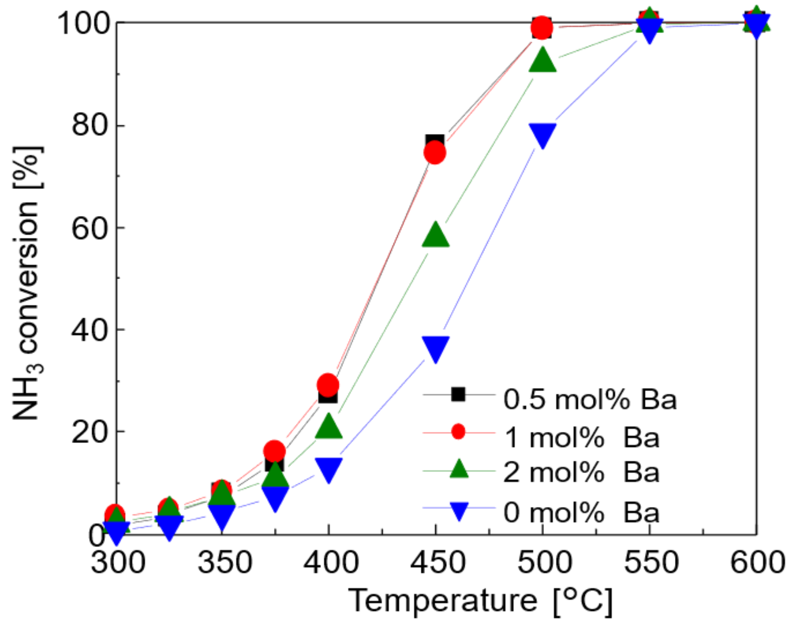

Figure S1. Influence of Ba amount on the activity of $Co/Ba_{0.01}Mg_{0.99}O$ catalyst. WHSV of 9000 mL g_{cat}^{-1} h^{-1}.

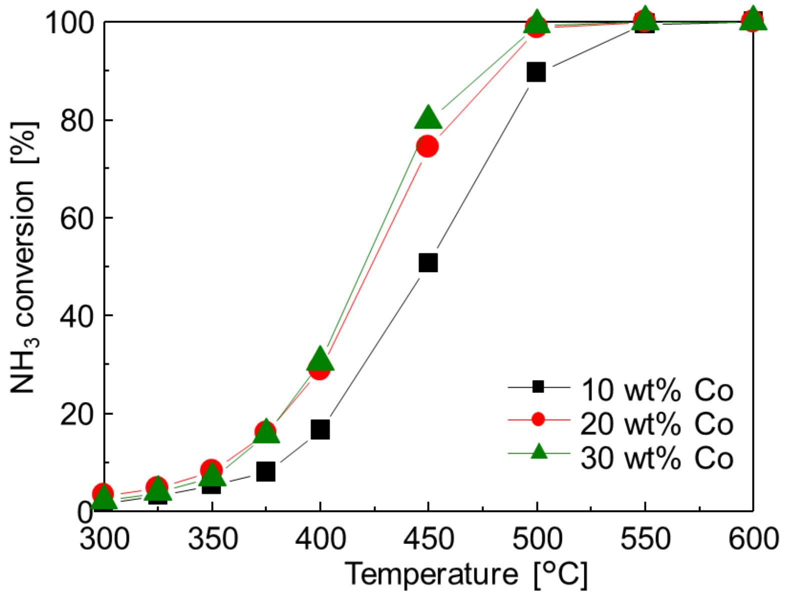

Figure S2. Influence of Co loading on the activity of Co/ $Ba_{0.01}Mg_{0.99}O$ catalyst. WHSV of 9000 mL g_{cat}^{-1} h^{-1}.

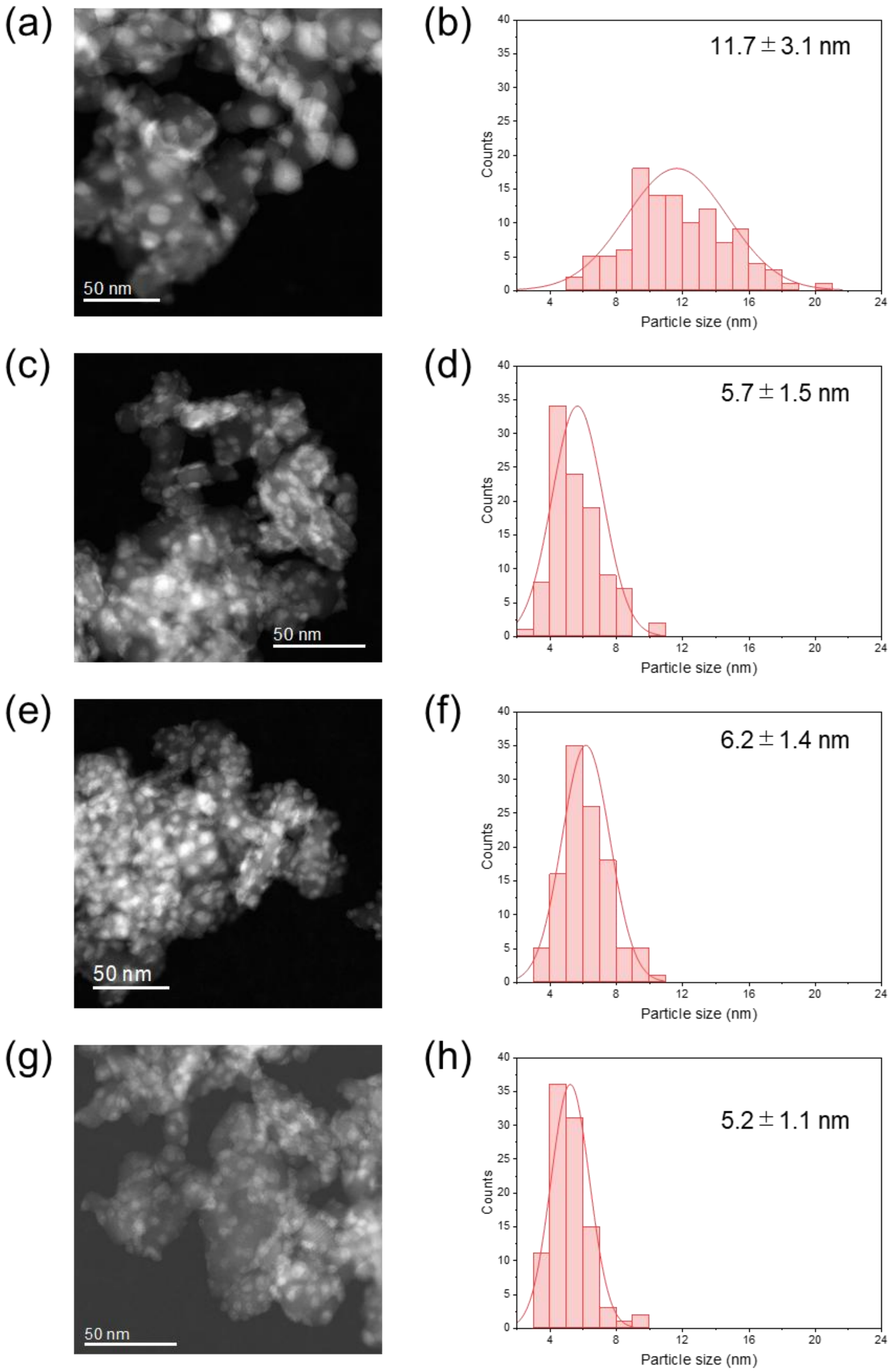

Figure S3. HAADF STEM images and Co nanoparticle distributions for Co/ $Ba_{0.01}Mg_{0.99}O$ (a–b), Co/ $Sr_{0.01}Mg_{0.99}O$ (c–d), Co/ $Sr_{0.01}Mg_{0.99}O$ (e–f), Co/MgO (g–h).

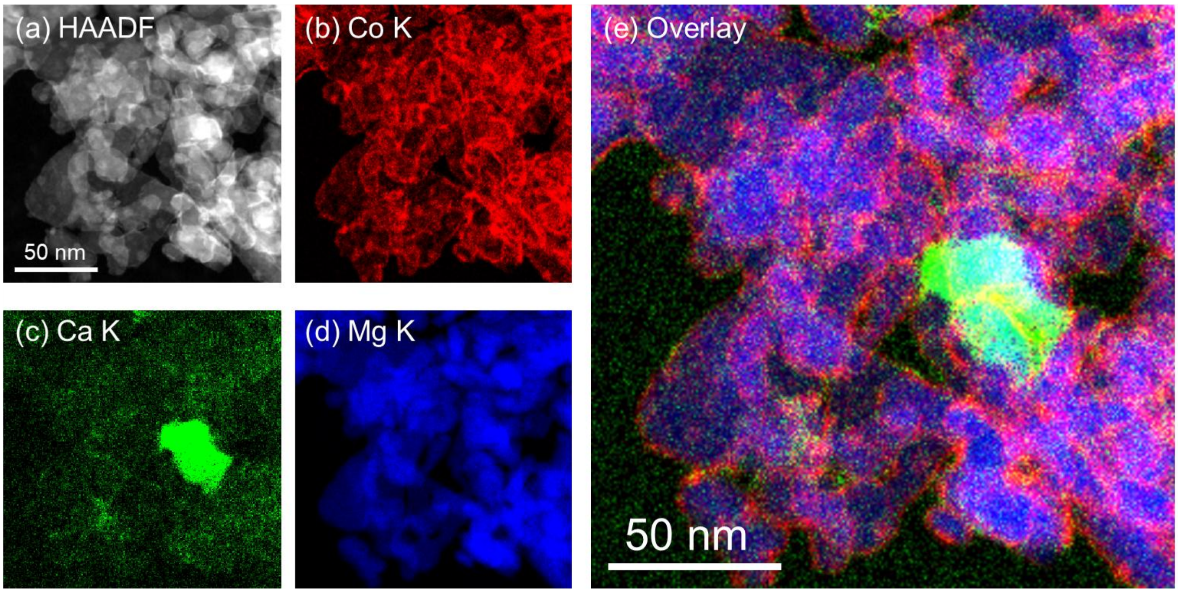

Figure S4. HAADF-STEM images and EDX maps of fresh Co/$Ca_{0.01}Mg_{0.99}O$ catalyst. (a) HAADF image. (b-e) EDX maps. (e) Overlay EDX map of (b) Co K, (c) Ca K, and (d) Mg K.

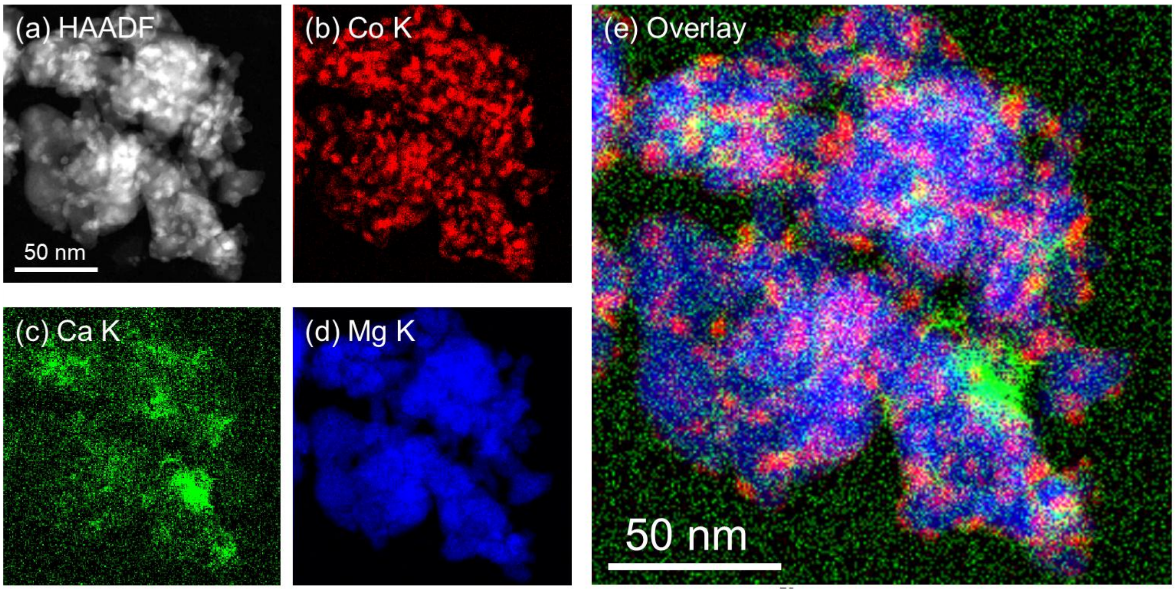

Figure S5. HAADF-STEM images and EDX maps of Co/$Ca_{0.01}Mg_{0.99}O$ catalyst after reduction. (a) HAADF image. (b-e) EDX maps. (e) Overlay EDX map of (b) Co K, (c) Ca K, and (d) Mg K.

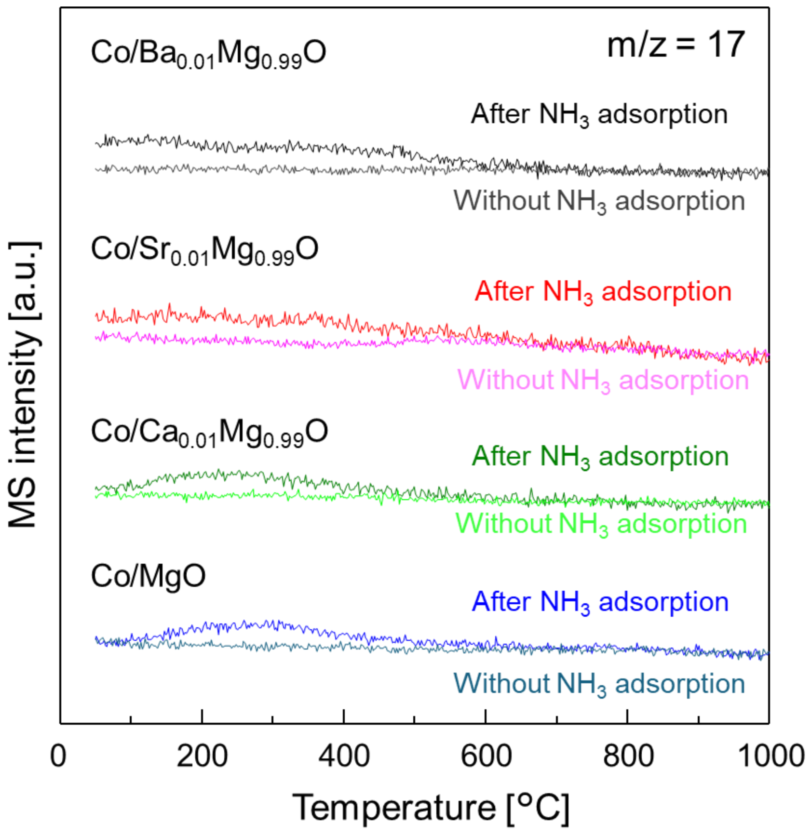

Figure S6. NH_3-TSR profiles of Co/$A_{0.01}Mg_{0.99}O$ (A = Ba, Sr, Ca) and Co/MgO after or without NH_3 adsorption at 400 °C. NH_3 (m/z = 17).

Table S1 Comparison of the catalytic activity of Co-based catalysts reported by other group

Catalysts	Co or Ni loading [wt%]	E_a [kJ mol^{-1}]	Temp. [°C]	WHSV [mL g_{cat}^{-1} h^{-1}]	NH_3 Conv. [%]	H_2 production rate [mol g_{cat}^{-1} h^{-1}]	Ref.
Co/$Ba_{0.01}Mg_{0.99}O$	20	53.4	500	9000	99.0	0.60	This work
			450		77.3	0.47	
			400		30.4	0.18	
		79.6	500	15,000	98.8	0.99	
			450		69.2	0.70	
			400		26.0	0.26	
		68.0	500	30,000	98.0	1.97	
			450		61.0	1.23	
			400		20.6	0.41	
		114.4	500	60,000	94.4	3.79	
			450		45.1	1.81	
			400		12.1	0.49	
Co/Sm_2O_3	8.2	85.7	550	15,000	87.3	0.88	4
20Co-10Ni/Y_2O_3	Co 12.91<br>Ni 6.53	67	550	30,000	71.2	1.43	25
$Ba_{0.4}$Co/C	8	69.4	500	14,000	100	0.94	31
Ni@MgO-NCNFs	10	-	450	30,000	99.4	2.00	35
$La_{0.5}Sr_{0.5}NiO_{3-\delta}-600H_2$	25.6	97.2	550	30,000	87.7	1.76	36
Ni/CaNH-HS	10	-	500	15,000	91.2	0.92	37
Ni/$MgAl_2O_4$-LDH	4.49	39.2	600	30,000	88.7	1.78	38
$Ni_7Y_{2.5}Al_{0.5}O_x$	47.7	118.7	500	30,000	100	2.01	39
Co/NC-600	34.4	75.16	500	30,000	80	1.61	40
35%Co/SiC-700	35	84.96	550	30,000	74.3	1.49	41
10Co/La(5)-Al_2O_3	9.12	71.6	550	9000	90	0.54	42
$LaCoO_x$/Co@NC/SBA-15(2D)	14.05	84.44	600	30,000	100	2.01	43
26Co-CAT(A)	25.8	31.82	500	6000	58	0.23	44
1.22 %Na-Co_3O_4	-	90.1	550	30,000	97	1.95	45
